# Interactions of endotoxin, albumin function, albumin binding capacity and oxidative stress in brain-dead organ donors

**DOI:** 10.1186/cc9944

**Published:** 2011-03-11

**Authors:** V Stadlbauer, B Leber, P Stiegler, S Stanzer, U Mayrhauser, S Köstenbauer, M Sereinigg, A Puntschart, T Stojakovic, K Tscheliessnigg, K Oettl

**Affiliations:** 1 University Hospital Graz, Austria; 2 Medical University Graz, Austria

## Introduction

Albumin binds and detoxifies endotoxin in healthy people. Oxidative stress leads to protein oxidation and thus to impaired binding properties of albumin. This, in combination with increased gut permeability, leads to appearance of endotoxin in the systemic circulation and further to impaired organ function. We hypothesise that these processes occur in serum of brain-dead organ donors.

## Methods

Eighty-four consecutive brain-dead organ donors were enrolled. Endotoxin was determined with an adapted limulus amoebocyte lysate assay. Albumin fractions and binding capacity were determined by HPLC. FlowCytomix™ was used for determination of cytokine levels and RT-PCR for analysis of tight junction protein (TJP) mRNA expression. Brain-dead organ donors were categorized by the length of ICU stay. Survival data of 76 organ recipients were collected.

## Results

Albumin binding capacity for dansylsarcosine was reduced in brain-dead organ donors compared with controls. Endotoxin positivity in was found in 16.7% of brain-dead organ donors. Endotoxin positivity but not length of ICU stay was associated with a further decrease of binding capacity. In organ donors albumin was higher oxidized than in controls. Lengths of ICU stay increased albumin oxidation further. In addition, IL-6, IL-8, IL-10 and IL-1β levels were elevated in patients whereas IFNγ levels were within the normal range. Recipients of organs from endotoxin-positive donors showed a significantly worse survival as compared with recipients from endotoxin-negative donors (log-rank *P *< 0.05). Length of ICU stay of the donor did not have any influence on outcome of the recipients. Preliminary data for TJP expression in duodenum samples showed a trend towards lower expression in the endotoxin-positive sample.

## Conclusions

We therefore conclude that oxidative stress, as well as systemic endotoxemia, is present in a proportion of in brain-dead organ donors that might have a negative impact on outcome of recipients. High endotoxin levels might be due to increased gut permeability and decreased binding capacity of albumin facilitated by higher albumin oxidation.

